# Metal Toxicity: Tattoos: Safe Symbols?

**Published:** 2005-09

**Authors:** Victoria McGovern

A 2003 Harris Poll reported that 16% of U.S. adults are tattooed, including over a third of those aged 25–29. Despite the art’s growing popularity, the toxicology of tattoos is poorly understood. Now some ink components—particularly heavy metals—have raised concerns. A lawsuit set to go to trial in October 2005 has been filed against nine tattoo ink companies for violations of California’s Proposition 65, which requires that Californians be warned before exposure to chemicals causing cancer, birth defects, or other reproductive harm.

“One reason we started looking at tattoos is that the research we’ve done suggests teenage girls in particular are a huge market now for tattoos,” says Deborah Sivas, president of the nonprofit American Environmental Safety Institute (AESI), which filed the suit. The concern is not that the inks are acutely harmful, but rather that chronic exposure to some metals—especially lead—is a known problem.

Titanium and aluminum are often used as colorants in tattoos; more worrisome, inks using nonmetal colorants may include traces of antimony, arsenic, beryllium, chromium, cobalt, lead, nickel, and selenium (AESI filed over the latter eight metals). Sivas says the ink used for a 3 by 5 inch tattoo contains 1–23 micrograms of lead, versus the 0.5 micrograms per day permitted under Proposition 65.

Understanding exposure to lead and other metals once incorporated into a tattoo is not simple. A healed tattoo is a complicated array of ink particles trapped within dermal fibroblasts, macrophages, and mast cells. “One of the biggest problems is, over the period of time, how is exposure evaluated?” says Westley Wood, president of Unimax Supply, a tattoo equipment supplier and ink producer, which settled out of court in the AESI lawsuit. “Should it be counted every single day for the rest of your life, or is it dissipated in the body within a month?”

“Metal toxicity has not been an observed problem,” asserts physician Linda Dixon, president of the American Academy of Micropigmentation, a cosmetic tattooing trade group and manufacturer of Kolorsource brand of cosmetic ink. However, she adds, “Information about pigments in traditional tattoo products is usually a trade secret and not shared. We need information which is scientifically based.”

Dixon suggests publishing a list of pigments that are known to be safe and those known to be toxic. “Know your colors, know your pigments,” she says. “The scientists know what to avoid, and this should be common knowledge in the tattoo industries.” Though tattoo inks are subject to regulation by the Food and Drug Administration as cosmetics and color additives, that agency does not currently attempt to actually regulate tattooing or the pigments involved.

Despite the upcoming court battle, among the 17% of tattooed Americans the Harris Poll say regret their indelible marks, the greatest reason for dissatisfaction is not the safety of the tattoo but having been inscribed with the wrong person’s name.

## Figures and Tables

**Figure:**
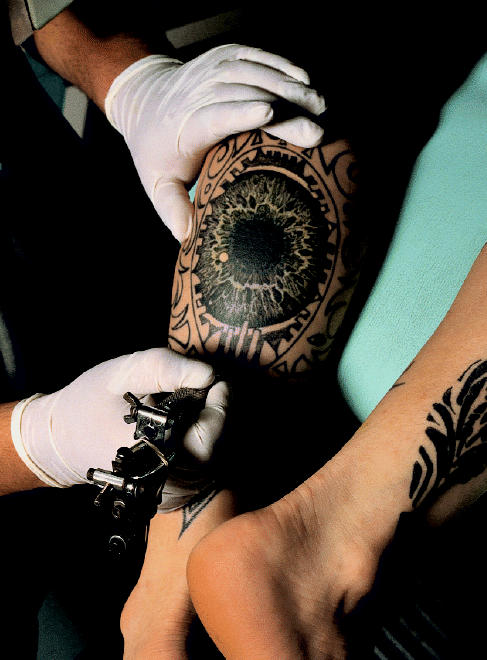
No clear picture. There are few health data for tattoo inks; with the growing popularity of the art, some see this as a cause for concern.

